# Accurate Vehicle Location System Using RFID, an Internet of Things Approach

**DOI:** 10.3390/s16060825

**Published:** 2016-06-04

**Authors:** Jaco Prinsloo, Reza Malekian

**Affiliations:** Department of Electrical, Electronics and Computer Engineering, University of Pretoria, Pretoria 0002, South Africa; jprinsloo613@gmail.com

**Keywords:** accurate vehicle location, GPS triangulation, GSM network, RFID, read range, passive transponder, database implementation

## Abstract

Modern infrastructure, such as dense urban areas and underground tunnels, can effectively block all GPS signals, which implies that effective position triangulation will not be achieved. The main problem that is addressed in this project is the design and implementation of an accurate vehicle location system using radio-frequency identification (RFID) technology in combination with GPS and the Global system for Mobile communication (GSM) technology, in order to provide a solution to the limitation discussed above. In essence, autonomous vehicle tracking will be facilitated with the use of RFID technology where GPS signals are non-existent. The design of the system and the results are reflected in this paper. An extensive literature study was done on the field known as the Internet of Things, as well as various topics that covered the integration of independent technology in order to address a specific challenge. The proposed system is then designed and implemented. An RFID transponder was successfully designed and a read range of approximately 31 cm was obtained in the low frequency communication range (125 kHz to 134 kHz). The proposed system was designed, implemented, and field tested and it was found that a vehicle could be accurately located and tracked. It is also found that the antenna size of both the RFID reader unit and RFID transponder plays a critical role in the maximum communication range that can be achieved.

## 1. Introduction

The concept known as the Internet of Things (*IoT*) is currently experiencing rapid growth in terms of the number of applications in which this concept can be applied. By extending the Internet and the Web into the physical realm, new opportunities are created and current existing challenges are addressed with sophistication and ease. The *IoT* concept refers to the incorporation of various technologies to existing products or scenarios, with the purpose of connecting those products to the Internet [1]. The result is that content and services would be available in physical areas, which opens the door to new opportunities. The *IoT* realm allows and promotes the interconnection of various smart devices in order to form an advanced computing environment. It should be noted that the present-day Internet infrastructure plays a vital role in achieving this interconnection. The development of the Internet model allowed for the interconnection of end-users through a communication medium. The main objective of the *IoT* field is to incorporate electronic systems into objects. This includes sensor nodes, communication platforms, and interactive display units. By doing so, a physical object can be developed into a “smart” object, which produces and consumes information [1]. The result is that mobile objects can also be connected to the Internet backbone. By interconnecting various smart objects, solutions to present-day challenges can be found. The *IoT* field provides support in localization and tracking capabilities, which is the focus of this article. Localization and tracking can be achieved with technologies such as GPS and GSM. These technologies have been used in a variety of initiatives in order to provide solutions to real-world limitations and solutions. These initiatives are discussed in Section 2.

Autonomous driving is considered to be an emerging concept that is rapidly evolving in the *IoT* realm. With the integration of various sensors to existing vehicle technology, driver assistance systems can be developed and implemented with the purpose of increasing road safety and driver awareness. Various autonomous systems are already present in modern-day vehicles, such as vehicle parking assistance systems and adaptive cruise control [2]. Challenges are however present when it comes to autonomous driving and vehicle positioning. A human driver has the inherent ability to make accurate route decisions based on given GPS information. Current GPS systems do however not provide the level of accuracy required for autonomous driver systems [3]. Furthermore, underground tunnels and dense urban areas limit GPS capabilities, which further complicates the concept of autonomous driving. The design of an accurate vehicle location system using RFID technology, as proposed in this article, will address the issues presented by modern-day infrastructure. Using the proposed system as a baseline, additional sensors such as gyroscopes or radar technology can also be added to increase the number sensor inputs, which in turn increases the localization accuracy [4]. This inevitably implies that autonomous driver assistance systems will receive more accurate information and this allows for better system autonomy.

The Global Positioning System (GPS) consists of a constellation of more than 20 satellites, which allows for accurate position triangulation [5]. GPS technology is primarily used in vehicles in order to facilitate route planning and vehicle position estimation. The primary requirement for effective GPS triangulation is direct line-of-site. Modern infrastructure, such as dense urban areas and underground tunnels, can effectively block all GPS signals, which implies that effective position triangulation will not be achieved. Multi-path effects could also be present, due to the high-rise buildings in dense urban locations [6]. In addition to the limitation listed above, an up-to-date map database is also a requirement for accurate vehicle location [6]. Several independent technologies, such as Global System for Mobile communication (GSM) and radio frequency identification (RFID) can however be integrated with GPS technology in order to overcome the limitations of a standalone GPS system.

The benefits of RFID technology can be seen in current areas where the technology is utilized. One of the benefits of an RFID transponder is that no line-of-sight is required and, more importantly, no human intervention is required [7]. This allows for better time management and better human resource utilization. Furthermore, the use of a unique ID allows for the identification of individuals or individual objects. Recent advances in the power consumption of electronic devices have allowed for the creation of small sensors used in medical applications, such as glucose sensors. The advances in the power consumption of devices also benefit the RFID arena. The creation of small transponders, with an operational distance of a few centimeters, can be used as medical implants for the purpose of storing an individual’s personal information. It is, therefore, clear that RFID technology, though an emergent technology, already has a significant impact on multiple fields. By incorporating this technology into the field of intelligent transport, a complete system can be created that allows for accurate tracking and monitoring in a wide variety of applications.

The primary objective of the research project is to make use of the various solutions and opportunities provided by the *IoT* realm in order to deliver a solution to a real-world challenge. As discussed above, smart objects can be created with the interfacing of additional electronic units such as sensors, actuators, and communication platforms. By utilizing the abilities of GPS technology, real-time location data can be retrieved on a periodic basis. GSM technology can be used as the communication platform for the system, which allows for the collected data to be stored in the cloud. RFID technology can be used as an additional sensing module that is used in complex environments (dense urban areas and underground tunnels). These standalone technologies are integrated into a single system that allowed for the locating and tracking of a vehicle in normal and complex environments, based on information that was retrieved and stored by the system. The three main subcomponents that are addressed are the acquisition of location data, the transmission and reception of location data, and the remote storage and analysis of vehicle location information. For this project, the RFID transponder was designed from first principles. One typical application for this system is the continuous tracking of cash-in-transit vehicles. Although the number of cash in transit robberies remained constant at a total of 145 for the 2013 to 2014 period [8], it is believed that these vehicles should always be monitored.

## 2. Related Works

One typical application where the integration of different independent technologies has been implemented is in the MITRA project (Monitoring and Intervention of the Transport of dangerous goods) [9]. The MITRA project was funded by the European Commission and the purpose of the project is to create, demonstrate, and test a system that allows for the monitoring of dangerous goods during the transportation phase. The need for this system arose from the fact that the European civil security authorities do not currently track vehicles that transports dangerous goods. This implies that no pre-emptive measures can be taken should a dangerous scenario arise [9]. The system relays real-time vehicle location information, as well as information about the goods that are currently being transported by the vehicles, to European civil security centers [9]. The main components of this system is an on-board terminal from which the position data is derived, and a GSM module that allows for the position information, as well as the vehicle ID, to be transmitted to a central server. The system allows European authorities to detect, and possibly prevent, dangerous scenarios. From this system it can be seen that various independent technologies can be integrated in order to form a complete system that addresses a specific need. The utilization of the cloud to store relevant data is also considered to be a significant tool in the development of an integrated system.

One possible solution to the enhancement of vehicle tracking is the development of an integrated system using GPS technology for tracking and GSM technology for the relaying of GPS coordinates to a central server and database system. A possible advantage of this solution is that multiple vehicle tracking systems can be installed in a collection of vehicles. This effectively allows for the monitoring and tracking of multiple vehicles, with data being managed from a central location. One initiative that is proposed is the development of an integrated GPS-GSM tracking system with the purpose of enhancing public transportation management services [10]. This system consists of a tracking system that is implemented on busses, a display system that is installed at bus stops, and a base station that facilitates the remote monitoring of data. The tracking system on the bus consists of a GPS receiver module and a GSM module, which allows the system to transmit the current location of the bus to the base station. The primary purpose of the base station is to process all bus information and to update all bus stop display systems with the latest bus position information. This allows commuters to actively be aware of the location of the busses that are relevant to specific bus stops. The different subcomponents of the proposed system will create a wireless network tracking system with the primary objective of facilitating the real-time tracking of public transport vehicles, hence simplifying the day-to-day movements and routines of commuters.

An initiative is also proposed for the development of a system that monitors the geographical location of a vehicle, as well as vehicle information such as the current vehicle speed [11]. The proposed system consists of a track and relay unit situated in the vehicle itself (GPS and GSM technology are once again integrated into a single system), as well as a remote server for the processing and analysis of data. The advantage of the proposed system is that effective law enforcement can be achieved. This system effectively allows for the remote monitoring of vehicle speeds. Based on the geographical position of the vehicle, it can be determined whether a vehicle is in direct violation of the speed limit. A full implementation of this system will remove the need for physical supervision in the field with regards to speed limit violations.

Another possible advantage of an integrated vehicle tracking system is that it can be used by large commercial organizations for the purpose of fleet management. An effective fleet management system can have a significant impact on the productivity and resource management of an enterprise [12]. The initiative proposed in [12] focuses on the development of a hybrid vehicle tracking system for the purpose of improving current fleet management systems and the distribution of police vehicles. The hybrid system consists primarily of GPS and GSM technology, as well as underlying software for the processing of data. It should be noted that GPS triangulation can be improved by implementing methods such as dead-reckoning and Kalman filtering [13].

The various initiatives discussed above greatly enhances the ability of vehicle tracking, which would otherwise not be possible with the use of a standalone GPS system. However, the primary challenge that is presented by complex environments still remains. In order to solve this challenge, the technology of radio frequency identification (RFID) is introduced. RFID is a wireless proximity communication method, which can be used as a standalone technology or it can be complementary to existing technologies [14]. RFID is present in a wide variety of applications. These applications include access control procedures, supply management, and protection against theft [14]. The field of RFID technology is also considered to be one of the fastest growing technology fields that exist today [15]. The main focus area of RFID technology in the past and present is security systems and access control methods. There has however been a rapid increase in the use of RFID systems in the transport sector, as well as in supply chain management infrastructures [15]. One of the predominant uses of RFID technology in the transport sector is in toll collection. The implementation of RFID readers on toll gate infrastructure, and the placement of transponders in vehicles, has proved to have a significant impact on the amount of traffic that can be processed by a toll gate. By implementing RFID technology in the toll system, traffic queues have been reduced and vehicle owners have been saved a considerable amount of time and effort. Animal tracking is also an area in which RFID technology has played a significant role. By placing transponders on animals, it is possible to remotely monitor the movements, and possibly even the behavior of animals [15].

Significant security concerns are however present. The wireless transfer of data provides an opportunity for potential attackers to exploit the system [15]. The benefits of RFID technology can be seen in the current areas where the technology is utilized. One of the benefits of an RFID transponder is that no line-of-sight is required and, more importantly, no human intervention is required [7]. This allows for better time management and better human resource utilization. Furthermore, the use of a unique ID allows for the identification of individuals or individual objects, which relates back to the presence of the technology in access control methods. A final implementation that will be discussed is the application of RFID technology in implantable medical devices. The size of an RFID transponder is determined by the specific application for which it is used. Recent advances in the power consumption of electronic devices have allowed for the creation of small sensors used in medical applications, such as glucose sensors [15]. The advances in the power consumption of devices also benefit the RFID arena. The creation of small transponders, with an operational distance of a few centimeters, can be used as medical implants, for the purpose of storing an individual’s personal information. There are however concerns about the reliability of RFID technology since RFID readers are known to provide unreliable data streams and, as in GPS signals, multipath issues are also present in RFID technology [16]. This has a direct effect in the tempo of acceptance of this technology in current widespread applications. Nevertheless, it is clear that RFID technology, though an emergent technology already has a significant impact on multiple fields. By incorporating this technology into the field of intelligent transport, a complete system can be created that allows for accurate tracking and monitoring in a wide variety of applications.

One proposed initiative is the development of an In-Transit Visibility system based on RFID technology [17]. The motivation for this initiative is based on the fact that export goods in Kenya are redirected to the local market, after which refund claims are made. The purpose of this system is to facilitate the tracking of export goods in order to ensure that they are indeed exported to the intended destination. An integrated tracking system is installed into the vehicle that ships the export goods. This system contains a GPS receiver, a GSM modem, and an RFID reader. The cargo doors of the vehicle are sealed with bolt electronic seals [17]. These seals are then interrogated by the RFID reader and the retrieved data, together with the GPS coordinates of the vehicle, are transmitted via the GSM network to a remote control room. The use of RFID technology can therefore be used in conjunction with other independent technologies in order to relay important information that would otherwise be unknown.

It is believed that the performance and functionality of applications, which focus on active vehicle-safety, rely heavily on real-time positioning [18]. The method that is proposed in [18], is to make use of RFID technology for the enhancement of road safety and traffic awareness. In this initiative, RFID transponders are placed on the road surface. The transponders are then interrogated by an RFID reader and the received data are exchanged with all nearby vehicles via wireless communication. After the exchange of data, the system can monitor the condition of traffic and issue alerts in dangerous situations. The method proposed in [18] can be modified to accurately locate and track vehicles in complex environments. This is considered to be the main motivation of the project. Since a standalone GPS system can operate effectively in an open environment, the utilization of RFID technology is only required in complex environments. RFID transponders are once again placed on the road surface. The RFID interrogator will then be integrated with the GPS receiver and GSM module in order to form a complete tracking system that can operate in both normal and complex environments. The RFID transponders contain a device-specific identification number, which will be used to determine the position of the vehicle. It is believed that South Africa would benefit from the implementation of accurate vehicle location systems, specifically systems that can locate and track the movement of a vehicle in any type of environment.

It is clear that the *IoT* field plays a predominant role in all of the above mentioned initiatives. The concept itself allows for the integration of various independent technologies in order to form a single, complete, interconnected system that addresses a wide variety of challenges.

## 3. System Overview

**Figure 1:**
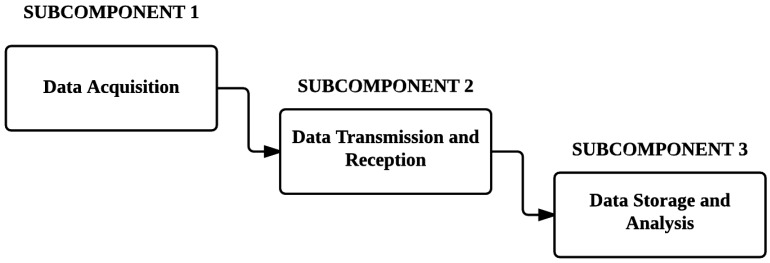
General overview of the desired system.

The main challenge that is addressed in this research project is accurate vehicle location and tracking in normal and complex environments, as well as the remote storage and analysis of vehicle information. Figure 1 illustrates an overview of the project concept:

With reference to the figure above, it is clear that three subcomponents can be identified, which are: the acquisition of location data; the transmission and reception of location data, and the remote storage and analysis of vehicle location information.

The proposed solution to the research project concept is to design and develop an integrated tracking system that makes use of various independent technologies in order to facilitate accurate vehicle location. Thus, the system consists of various modules, such as a GPS receiver module, an RFID reader module, and a GSM network module. The purpose of the GPS receiver module is to accurately triangulate the position of the vehicle in a normal environment. The RFID reader module interrogates RFID transponders situated on the road surface. This facilitates vehicle location in a complex environment. It should be noted that interference would not be a problem since the reading range is limited to the distance between a single vehicle and a transponder. The purpose of the GSM module is to facilitate wireless communication to a remote station. The remote station can obtain access to a dedicated cloud server, in which all data is stored and can be analyzed remotely. This station stores and displays the vehicle information with the use of a database system and graphical user interface.

The use of GPS is considered to be the preferred method for real-time vehicle positioning [18]. A GPS system alone is however not entirely accurate (an accuracy of approximately 15 to 30 m can be achieved by GPS systems on navigation devices and consumer electronics [13]). It is, therefore, decided to investigate and consider various methods for improving GPS accuracy.

A wide range of wireless communication technologies exist, such as Bluetooth, ZigBee radio, Wi-Fi, and GSM. All of the technologies mentioned above have advantages and disadvantages however, due to the nature in which the system is implemented; we take advantage of the GSM technology. The other communication technologies mentioned above can however be used in indoor localization.

RFID consists of two main units. The first unit is the reader module or interrogator and the second unit is the transponder or tag. The purpose of the interrogator is to wirelessly retrieve data that is stored on the transponder by means of inductive coupling. In this research, the transponder is designed by doing research on the appropriate reading range necessary for this research work. Two design considerations are present with regards to transponder design: an active transponder design, or a passive transponder design.

An active tag design allows for the interrogator to have a built-in power source. This allows for a dramatic increase in the interrogation distance, but a significant increase in the cost of transponder production [15]. A passive tag design does not include a built-in power source; hence the tag relies solely on the reader module to provide the necessary power. In a passive transponder design, the maximum interrogation distance that can be achieved will be less compared to active transponders however, the production costs are also significantly reduced [15]. The different frequency bands in which tags can operate should also be considered. RFID tags can operate in the following frequency bands [15]: Low frequency (125–134 kHz), High frequency (13.56 MHz), Ultra high frequency (865–868 MHz), and Microwave (2.45 GHz).

Lower frequencies such as the low frequency and high frequency ranges allow for the design of passive transponders. Inductive coupling is considered to be the method of choice for power transfer and data transmission for tags in lower frequencies. When higher frequency transponders are desired, active transponders are used. In this design, we concluded that a low frequency passive transponder will be sufficient due to required reading range. The design procedure is followed in Section 4.

The third subcomponent of the system is the remote storage and analysis of vehicle location information. The storage of data is facilitated through the use of a database system. The data is graphically displayed to the user for analysis by means of a graphical user interface (GUI). The GUI is linked to the database system and data is retrieved by the execution of an HTTP web request. The GUI itself is in the form of a desktop application. It is decided that the desktop application will be developed with the use of the Microsoft NET platform, due to the fact that almost all computer systems support Windows .NET applications.

## 4. System Design

### 4.1. Main System Design

The main system consisted of three independent modules that is controlled by a microcontroller. The three modules included a GPS module, a GSM module, and an RFID reader module. Each module required an independent communication platform in order to provide the required data. It is, therefore, decided to make use of a 16-bit microcontroller with enough serial communication peripherals. The PIC24FJ64GB202 microcontroller is used as the main control unit of the system. Each module is interfaced to the microcontroller and the controller itself is given full control of the operation of each module.

(1) RFID reader module: The RFID reader module is an off-the-shelf product. In order to achieve a significant read range, attention should be given to both the reader and transponder antennas. It is, therefore, decided to make use of a reader that allowed for the interfacing of an external antenna (to improve the reading range of passive RFID transponder and improve accuracy of our systems [19,20], an external antenna is provided with the reader). The specifications of the RFID reader module, obtained from the technical documentation, can be viewed in Table 1 below.

It is required to calculate the voltage that developed across the antenna, as well as the current that flowed through the antenna. This is done with the following two equations, which is also obtained from the technical documentation [21].

(1) $$V_{\text{pp}}=\frac{2}{f_{0}C_{\text{tune}}\left(R_{t}+6\right)}$$

(2) $$I_{\text{ant}}=\frac{6.37}{R_{t}+6}$$

where $V_{\text{pp}}$ is the peak-to-peak voltage across the reader antenna, $f_{0}$ is the operating frequency of the reader, $C_{\text{tune}}$ is the value of the tuning capacitor of the reader, $I_{\text{ant}}$ is the current flowing through the antenna, and $R_{t}$ is the value of the internal resistance of the antenna, as well as any external resistance added in series with the antenna. The module already contains an internal resistance of 22 Ω, and an internal tuning capacitance of 532 pF. It is decided to add an additional tuning capacitor with a value of 100 pF. This would give a total value of 632 pF for the tuning capacitance. The inductance of the reader antenna is measured to be approximately 2.7 mH, and the antenna also had an internal resistance of approximately 9 Ω. By using the inductance of the reader antenna and the tuning capacitance, the frequency of operation is determined with the use of Equation (3) below:

(3) $$f_{0}=\frac{1}{2\pi \sqrt{L_{R}C_{R}}}$$

where $L_{R}$ is the inductance of the reader antenna and $C_{R}$ is the total tuning capacitance.

From Equation (3):

$$\begin{array}{l} f_{0}=\frac{1}{2\pi \sqrt{L_{R}C_{R}}}\\ \Rightarrow f_{0}=\frac{1}{2\pi \sqrt{\left(2.7\times 10^{-3}\right)\left(632\times 10^{-12}\right)}}\\ =121.8\text{ kHz} \end{array}$$

From Equation (1), the voltage is then calculated as follows:

Vpp=2f0Ctune(Rt+6) ⇒Vpp=2(121.8×103)(632×10−12)(9+22+6)  =702 V

The voltage calculated above is reduced to the maximum value stated in the module specifications. By using Equation (1), it is determined that an external resistor with a value of 100 Ω should be added, in order to adjust the voltage to a value that is within the required specifications. The total series resistance is therefore 131 Ω. From Equation (2), the current is then calculated:

$$\begin{array}{lll} & I_{\text{ant}} & =\frac{6.37}{R_{t}+6}\;\\ \Rightarrow & I_{\text{ant}} & =\frac{6.37}{\left(131\right)+6}\;\\ & & =46\text{ mA} \end{array}$$

The current value is well below the maximum rating specified in the specifications.

(2) GPS and GSM module: The GPS module contained an integrated GSM module as well. This allowed for the use of only one serial communication channel between the control unit and the module. Through this channel, both GPS and GSM functionalities could be accessed. Attention (AT) commands are used to facilitate communication with the module. The module itself required a separate supply voltage of 4 V and as mentioned above, a separate LiPo battery is incorporated to provide the necessary power. The module contains two receiver antennas: one antenna serves the purpose of receiving GPS signals, and the other antenna allows for an uplink to the GSM network. MTN South Africa is considered to be the service provider of choice. Table 2 below gives an overview of the AT commands that are used in software for general module communication. All AT commands are stored on the microcontroller and this therefore allowed the control unit to have full control over the module.

**Figure 2:**
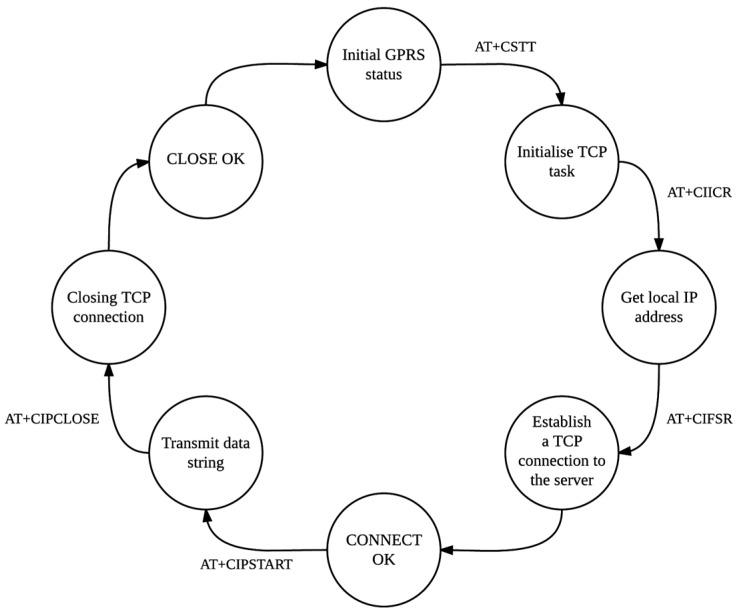
State machine showing the different states of the GSM connection process.

The state machine in Figure 2 above has been implemented on the control unit and is executed regardless of whether the system is in RFID mode or GPS mode. The only difference between the RFID mode and the GPS mode with regards to the wireless transmission of data, is the data string itself (in RFID mode, a tag ID is presented and not GPS data).

(3) Database system and Graphical User Interface: The database system is implemented with the use of the PHP scripting language. As mentioned above, all data strings are in the form of a POST command. The server received the transmitted strings via the established TCP connection and updated the database fields with the retrieved information. The transmitted data consisted of a vehicle registration number, the longitude coordinates, the latitude coordinates, the longitude indicator (North or South), the latitude indicator (East or West), and a timestamp. For the RFID transponder, the data string consisted of only a vehicle registration number, a transponder ID, and a timestamp. The transponder ID is considered to be linked to a specific GPS coordinate set, which is also stored in the database. The purpose of the GUI is to retrieve the stored data in the database to determine and plot a route by use of use of a Google Maps interface.

A Kalman filter is incorporated into the design of the interface in order to offer the application some level of prediction. The filter is only considered as a tool in the broader picture of the vehicle estimation algorithm. The Accord.NET math library is also used to assist with this task.

### 4.2. RFID Transponder Design

The main objective is to design and implement the RF front-end of the RFID transponder. This consists of the design and implementation of an LC tank that resonates on the carrier frequency of the RFID reader module. The resonant frequency is once again determined by Equation (3). Extensive simulations have been conducted on different antenna sizes and parameters. The purpose of the simulations is to obtain a relationship between the different antenna parameters and the induced voltage, measured at different load values. The internal parameters that are present in the LC tank are listed in Table 3 [22].

The coil inductance and coil capacitance is considered to be the two crucial parameters, due to the fact that these parameters directly affect the frequency of operation of the transponder, based on Equation (1). The internal capacitance of the coil is not enough to allow for resonance at the frequency of operation. It is, therefore, required to add additional capacitance to the coil. This additional capacitance, together with the inherent capacitance of the coil and the parasitic capacitance present in the internal circuitry of the transponder, forms an LC tank.

It is found that an increase in the antenna diameter, as well as an increase in the number of windings of the antenna, leads to an increase in the induced voltage across the antenna coil.

1) Antenna simulations and hardware design: They are two options for the best practical implementation of the transponder. The first design choice is to make use of a circular shaped antenna, due to the fact that most off-the-shelf passive tags today has an internal circular coil. The equation for the inductance of an N-turn multilayer circular coil is as follows [22]:
  (4) $$L=\;\frac{0.31{\left(\text{aN}\right)}^{2}}{6a+9\text{ha}+b}$$
  where L is the inductance of the coil in μH, a is the average radius of the coil in centimeters, N is the number of windings of the coil, h is the height of the coil windings in cm, and b is the thickness of the coil windings in cm. Table 4 shows the design parameters of the circular coil.

The antenna parameters in the table above are chosen such that a high quality factor can be obtained. By keeping the internal resistance as low as possible, a high quality factor can be obtained, as is discussed in the theoretical background section. The inductance of the coil can now be calculated with the use of Equation (4):

$$\begin{array}{ll} L & =\;\frac{0.31{\left(\mathrm{aN}\right)}^{2}}{6a+9h+10b}\;\\ & =\;\frac{0.31{\left(16\times 60\right)}^{2}}{6\left(16\right)+9\left(0.2\right)+10\left(0.2\right)}\;\\ & =\frac{0.31\left(921600\right)}{99.8}\;\\ & =2.862\;\mathrm{mH} \end{array}$$

Using Thompson’s Equation (3), the capacitance can be calculated which would allow for resonance to be achieved on the desired frequency of 129 kHz.

$$\begin{array}{cc} f_{0} & =\frac{1}{2\pi \sqrt{\mathrm{LC}}}\;\\ \Rightarrow 129\times 10^{3} & =\frac{1}{2\pi \sqrt{\left(2.862\times 10^{-3}\right)C}}\;\\ \Rightarrow C & ={\left(\frac{1}{2\pi \left(129\times 10^{3}\right)}\right)}^{2}÷\left(2.862\times 10^{3}\right)\\ & =531.85\times 10^{-12}\;\\ & =531.85\;\mathrm{pF} \end{array}$$

After the calculation of the required capacitance, the next step is to determine the theoretical internal resistance of the coil. As discussed in the theoretical background section, two types of resistances are present: a DC resistance value and an AC resistance value. The DC resistance for the circular loop antenna will now be calculated:

$$\begin{array}{ll} R_{\mathrm{DC}} & =\frac{l}{\sigma \pi a^{2}}\;\\ & =\frac{30.385}{\pi \left(5.8\times 10^{7}\right){\left(100\times 10^{-6}\right)}^{2}}\;\\ & =16.675\;Ω \end{array}$$

In order to calculate the AC resistance of the coil, the skin depth for copper wire at a frequency of 129 kHz should first be calculated:

δ=1πf0μσ=1πf0μ0μrσ=1π(129×103)(4π×10−7)(1)(5.8×107)=0.1839 mm

The AC resistance of the coil can now be calculated from the approximated AC resistance equation:

RAC≈12πaδσ≈30.3852π(100×10−6)(186×10−6)(5.8×107)≈30.3856.7783≈4.48 Ω

The total amount of ohmic losses is the sum of the values of the DC resistance and AC resistance of the coil. It should be noted that the internal radiation resistance of the coil can be considered as negligible.

RT=RDC+RAC=16.675+4.48=21.15 Ω

With the total theoretical internal resistance calculated, the quality factor (Q-factor) of the coil can now be determined:

$$\begin{array}{l} Q=\frac{Energy_{stored}}{Energy_{dissipated}}\\ =\frac{\omega L}{r}\\ =\frac{2\pi f_{0}L}{r}\\ =\frac{2\pi \left(129\times 10^{3}\right)\left(2.862\times 10^{-3}\right)}{21.15}\\ =109.6 \end{array}$$

**Figure 3:**
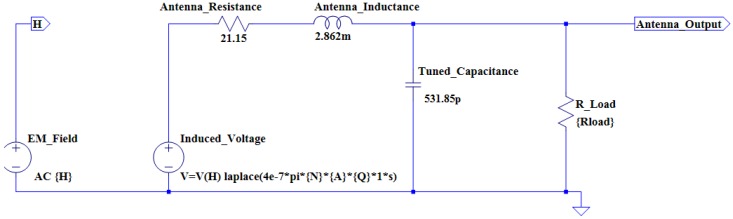
The circular antenna simulation model.

From the calculations above, it is possible to construct an antenna model using the LTSpice IV simulation software. Figure 3 illustrates the antenna model for the circular coil antenna.

The second antenna design option is use of a square shaped antenna. The mathematical calculations for the antenna parameters are considered to be more complex. A different shape is considered in order to determine and compare the performance of two geometrically different coils. Furthermore, it is also decided to increase the geometrical dimensions of the square loop coil. It is decided to reduce the theoretical inductance value of the second coil to 1 mH, in order to determine whether a relationship exists between two different inductance values. The equation for the inductance of an N-turn multilayer square coil is as follows [22]:

(5) $$L=0.008aN^{2}\left\{2.303\log_{10}\left(\frac{a}{b+c}\right)+0.2235\left(\frac{b+c}{a}\right)+0.726\right\}\left(\mu H\right)$$

where N is the number of windings, a is the distance between the center of the square coil and the outer edge of the coil windings, b is the width of the coil winding, and c is the height of the coil winding. Table 5 shows the design parameters for the square loop antenna.

Using Equation (5), and assuming an inductance value of 1 mH, the number of windings for the square coil can be calculated:

$$\begin{array}{l} L=0.008{\text{aN}}^{2}\left\{2.303\log_{10}\left(\frac{a}{b+c}\right)+0.2235\left(\frac{b+c}{a}\right)+0.726\right\}\\ \Rightarrow 1000=0.008\left(13\right)N^{2}\left\{2.303\log_{10}\left(\frac{13}{0.3+0.3}\right)+0.2235\left(\frac{0.3+0.3}{13}\right)+0.726\right\}\\ \Rightarrow 1000=0.104N^{2}\left\{2.303\log_{10}\left(\frac{65}{3}\right)+0.2235\left(\frac{3}{65}\right)+0.726\right\}\\ \Rightarrow 1000=0.104N^{2}\left\{3.813\right\}\\ \Rightarrow 1000=0.3965N^{2}\\ \Rightarrow N=50.22\\ \Rightarrow N\approx 51 \end{array}$$

Using Equation (3), the capacitance can again be calculated that would allow a resonance to be achieved at a frequency of 129 kHz. Since the inductance is chosen to be 1 mH, the tuning capacitance should be recalculated:

$$\begin{array}{cc} f_{0} & =\frac{1}{2\pi \sqrt{\text{LC}}}\\ \Rightarrow 129\times 10^{3} & =\frac{1}{2\pi \sqrt{\left(1\times 10^{-3}\right)C}}\\ \Rightarrow C & ={\left(\frac{1}{2\pi \left(129\times 10^{3}\right)}\right)}^{2}÷\left(1\times 10^{-3}\right)\\ & =1.52\times 10^{-9}\;\\ & =1.52\text{ nF} \end{array}$$

The next step is to determine the theoretical internal resistance of the coil. Both the DC resistance and AC resistance needs to be recalculated for the square antenna due to the fact that the geometric parameters differ from that of the circular coil:

$$\begin{array}{ll} R_{\text{DC}} & =\frac{l}{{\sigma \pi a}^{2}}\\ & =\frac{8\text{aN}}{{\sigma \pi a}^{2}}\\ & =\frac{8\left(0.13\right)\times 51}{\pi \left(5.8\times 10^{7}\right){\left(200\times 10^{-6}\right)}^{2}}\\ & =7.27\;Ω \end{array}$$

The AC resistance of the coil can be calculated from the approximated AC resistance equation. It should be noted that the skin depth stays constant for both antennas, since both antennas will be constructed from copper wire and both will operate at a frequency of approximately 129 kHz:

$$\begin{array}{ll} R_{\text{AC}} & \approx \frac{l}{2\pi a\delta \sigma }\\ & \approx \frac{8\text{aN}}{2\pi a\delta \sigma }\\ & \approx \frac{8\left(0.13\right)\times 50}{2\pi \left(100\times 10^{-6}\right)\left(186\times 10^{-6}\right)\left(5.8\times 10^{7}\right)}\\ & \approx \frac{7.82}{6.7783}\\ & \approx 1.154\;Ω \end{array}$$

The total amount of ohmic losses are as follows:

$$\begin{array}{ll} R_{T} & =R_{\text{DC}}+R_{\text{AC}}\\ & =7.27+1.154\\ & =8.43\;Ω \end{array}$$

With the total theoretical internal resistance calculated, the quality factor (Q-factor) of the coil can now be determined.

$$\begin{array}{ll} Q= & \;\frac{\text{Energy stored}}{\text{Energy dissipated}}\\ & =\;\frac{\text{reactance}}{\text{resistance}}\\ & =\;\frac{\omega L}{r}\\ & =\frac{2{\pi f}_{0}L}{r}\\ & =\;\frac{2\pi \left(129\times 10^{3}\right)\left(1\times 10^{-3}\right)}{8.43}\\ & =96.14 \end{array}$$

**Figure 4:**
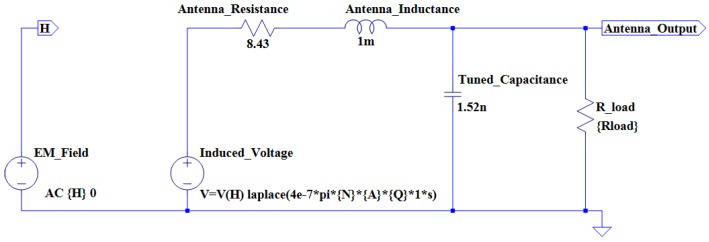
The square antenna simulation model.

An antenna model is once again constructed in LTSpice IV. Figure 4 illustrates the antenna model for the square coil.

2) Transponder power supply circuitry: The designed LC tank provides two important abilities to the RFID transponder. The first ability is that the transponder can draw the required power for effective operation from the RF-field. As mentioned above, this power is made available by the RFID reader module. The second ability is that the transponder can effectively communicate with the RFID reader [23] module through the RF-field. This allows for the transfer of the transponder ID to the reader module.

**Figure 5:**
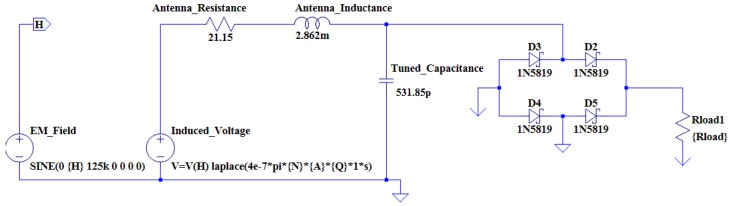
The simulation model of the LC tank and rectifier circuit.

The next step in the design process is to design, simulate and implement a power supply system for the transponder. The power supply system receives an AC power signal from the LC tank. This signal should be converted into a stable DC power signal in order to power the internal circuitry of the transponder. It is decided to implement a full-wave bridge rectifier for the rectification stage of the power supply. The full-wave bridge rectifier allows for the passage of current in one direction only during a full AC cycle. During the positive half cycle, diodes D1 and D3 is forward biased and diodes D2 and D4 is reversed biased. For the negative half-cycle, D1 and D3 is reverse biased and D2 and D4 is forward biased. In order to achieve a maximum interrogation range, the power consumption of the transponder should be as low as possible. It is, therefore, decided to make use of Schottky diodes due to the fact that they have a minimal forward voltage drop (a forward voltage drop of approximately 0.2 V is measured during the implementation phase of the transponder). Figure 5 shows the simulation model of the LC tank and rectifier circuit:

The next phase is to filter and smooth the rectified signal in order to provide a clean and stable DC supply to the rest of the transponder electronics. This is done with the addition of filter capacitors after the rectification stage. Equation (6) can be used to calculate the value of the filter capacitors [24]:

(6) $$v_{r}=\frac{V_{\text{cmax}}}{2f_{0}R_{L}C}$$

where $v_{r}$ is the ripple voltage, $V_{\text{cmax}}$ is the maximum input voltage, $f_{0}$ is the frequency of the input signal, $R_{L}$ is the load value, and C is the filter capacitor value.

Since the frequency of the input voltage is between 125 kHz and 134 kHz, the overall ripple voltage will remain significantly small. Since a microcontroller is used for the software part of the transponder, the filter capacitance value is chosen to be relatively high, in order to reduce the external effects that a high internal oscillator might have on the system. Three separate capacitors are incorporated into the design: a 470 µF electrolytic capacitor, a 22 µF electrolytic capacitor and a 100 nF ceramic capacitor. If it is assumed that the maximum input voltage is 20 V, the ripple voltage for a 50 kΩ load can be calculated as follows:

$$\begin{array}{c} v_{r}=\frac{V_{\text{cmax}}}{2f_{0}R_{L}C}\\ v_{r}=\frac{30}{2\left(125\times 10^{3}\right)\left(50000\right)\left(492.1\times 10^{-6}\right)}\\ \Rightarrow v_{r}=\frac{30}{6.15125\times 10^{6}}\\ \Rightarrow v_{r}=4.877\;µV \end{array}$$

The final design phase in the transponder power supply unit is to design and implement a voltage regulator circuit in order to limit the supply voltage of the transponder to the desired operating voltage of the internal circuitry. If the distance between the transponder and reader is decreased, the coupling coefficient is increased, which leads to a dramatic increase in the induced voltage over the transponder antenna. In order to limit the supply voltage, the approach described in [15] is followed, which involved the design of a shunt regulator circuit with the use of a transistor and Zener diode. Equation (7) shows how the value of the shunt resistor can be calculated [15], if the value of the load, as well as the coupling coefficient is known:

(7) $$R_{s}=\left|\frac{1}{\frac{\left(\frac{j\omega k\sqrt{L_{1}L_{2}}i_{1}}{v_{u}}\right)-1}{{j\omega L}_{2}+R_{2}}-{j\omega C}_{2}-\frac{1}{R_{L}}}\right|$$

where k is the coupling coefficient between the reader and transponder, $L_{1}\;$ is the inductance of the reader antenna, $L_{2}$ is the inductance of the transponder antenna, $i_{1}$ is the current flowing through the reader antenna, $R_{2}$ is the internal resistance of the transponder coil, $C_{2}$ is the capacitance value of the tuning capacitor, and $R_{L}\;$ is the load resistance [15]. Typical resistance values of the shunt regulator are in the order of a few hundred ohms to one kilo ohm [15]. Since the voltage would be very low at a reading distance of 25 cm to 30 cm, the use of a perfect shunt regulator is not really required. It is, however, still implemented in order to provide protection at smaller distances. Once the Zener breakdown voltage has been reached, the Zener will start to conduct and the base of the transistor will switch on. Current will then flow through the regulator circuit, which limits the voltage.

**Figure 6:**
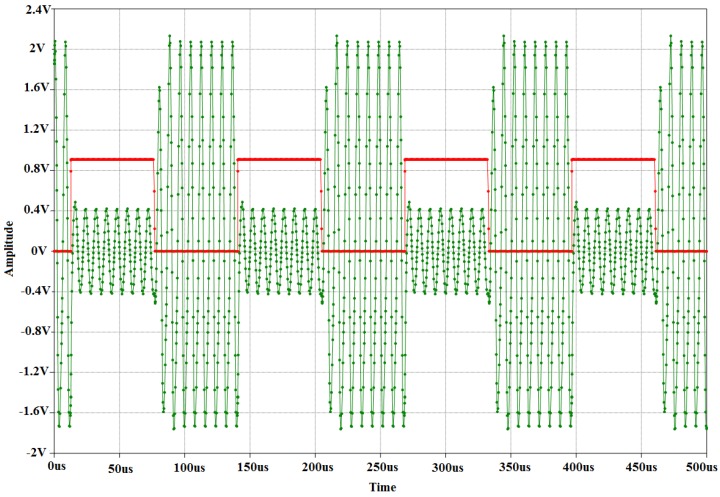
The transient analysis of the load modulator simulation.

3) Transponder load modulation circuitry: The next phase in the design process is to design, simulate, and implement the load modulation circuit. The purpose of the load modulation circuit is to manipulate the RF-field between the reader and transponder, in order to transmit data from the transponder to the reader. The load modulator consists of a resistor and a switch. The resistor is directly connected to the LC tank. The purpose of the switch is to connect or disconnect the resistor to or from the antenna. By doing so, the parameters of the transponder resonant circuit are changed, which results in variations in the magnitude and phase of the transponder impedance (modulation). The variations can be detected by the reader through the RF-field, and by applying an appropriate procedure, the reader can reconstruct (demodulate) the transmitted data. The switch is implemented with the use of a FET, due to the fact that FETs are used in the construction and fabrication of logic switches in the field of microelectronics. A PIC microcontroller is used as the data carrier, and the transmission pin of the data carrier is directly connected to the gate of the FET. This allows the data carrier to have full control over the modulation. The source of the FET is connected to the resistor which, in turn, is connected to one of the resonant circuit terminals. Figure 6 shows the simulation model for the load modulator. A simple clock divider circuit is implemented in order to simplify the simulation of load modulation. The purpose of the clock divider is to simulate the real-time transmission of data from the microcontroller. The EM4100 RFID communication protocol has been implemented on the transponder. This implies that data bits are transmitted at a rate of 2 kHz. In the simulation, a sine wave generator is used to simulate the induced voltage in the resonant circuit. The resonant circuit is represented by an inductor and capacitor. The load modulator is connected to the resonant circuit and receives a square wave input from the clock divider. Figure 6 shows the transient analysis of the load modulator simulation.
4) Transponder data carrier: The transponder resonant circuit, load modulator, and power supply unit have been designed and implemented in hardware, as discussed above. The hardware components allow for the harnessing of power and the transmission of data. Next, in the transponder design is to implement the data carrier. The data carrier serves the purpose of transmitting the digital ID of the transponder. An ID, consisting of 10 hexadecimal values for compliance to the EM4100 RFID communication protocol, is chosen and converted to the corresponding binary values. A software routine is implemented on the microcontroller to transmit the corresponding bit values. Transmission is done by controlling the voltage of one of the microcontroller pins (a high pin voltage corresponds to the transmission of a binary one and a low pin voltage corresponds to a binary zero). The main constraint in the transponder design is the amount of power available. It is, therefore, decided to use a low-power microcontroller for the data carrier.

The design consists of the transmission of nine header bits. The header bits are all logical high bits. The second phase consists of the transmission of the actual data bits. A total of 10 hexadecimal values are transmitted, which equates to a total of 40 bits. The EM4100 protocol makes use of even parity for data validation purposes. In addition to the 40 data bits, each hexadecimal value receives a parity bit, which results in a total of 5 bits per hexadecimal value. With the parity bits included in the transmission, the total number of data bits is 50. The last phase is to transmit a column parity bit combination, as well as the stop bit. The column parity combination consists of 4 parity bits. The total number of bits that are transmitted is therefore 64. The transmission of data will be repeated as long as the transponder has sufficient power to operate.

The data bits cannot be directly transmitted. It is required to first encode the transmission bits before activating the load modulator. The general line encoding scheme that is used in low-frequency RFID transponders is Manchester encoding. Manchester encoding can be implemented as hardware architecture, or as a software routine. The hardware implementation requires the use of an XOR gate. The transmission rate clock and the data itself serves as inputs to the gate and the resulting output, which is the Manchester encoded data, is connected to the gate of the load modulator switch. Two software routines are implemented, one routine for a logical high and another routine for a logical low. The data is directly encoded in software which implies that the output pin of the microcontroller can directly be connected to the load modulator. The two routines consisted of a logical high or low value, followed by a delay routine. The EM4100 requires a transmission rate of 2 kHz (64 carrier frequency clock cycles) or 4 kHz (32 carrier frequency clock cycles) when a carrier frequency of 125 kHz is used. A transmission rate of 64 clock cycles is chosen, which implies that a delay of 32 clock cycles would be required to encode the data with the Manchester encoding scheme. Since the RFID reader module is configured to operate at a carrier frequency of 129 kHz, the time delay required for the Manchester encoding scheme can be calculated from Equation (8):

(8) $$\begin{array}{l} T_{\text{delay}}=\frac{1}{\frac{f_{0}}{32}}\\ \Rightarrow T_{\text{delay}}=\frac{1}{\frac{129\times 10^{3}}{32}}\\ =\frac{1}{4.031\times 10^{3}}\\ =248\;µs \end{array}$$

**Figure 7:**
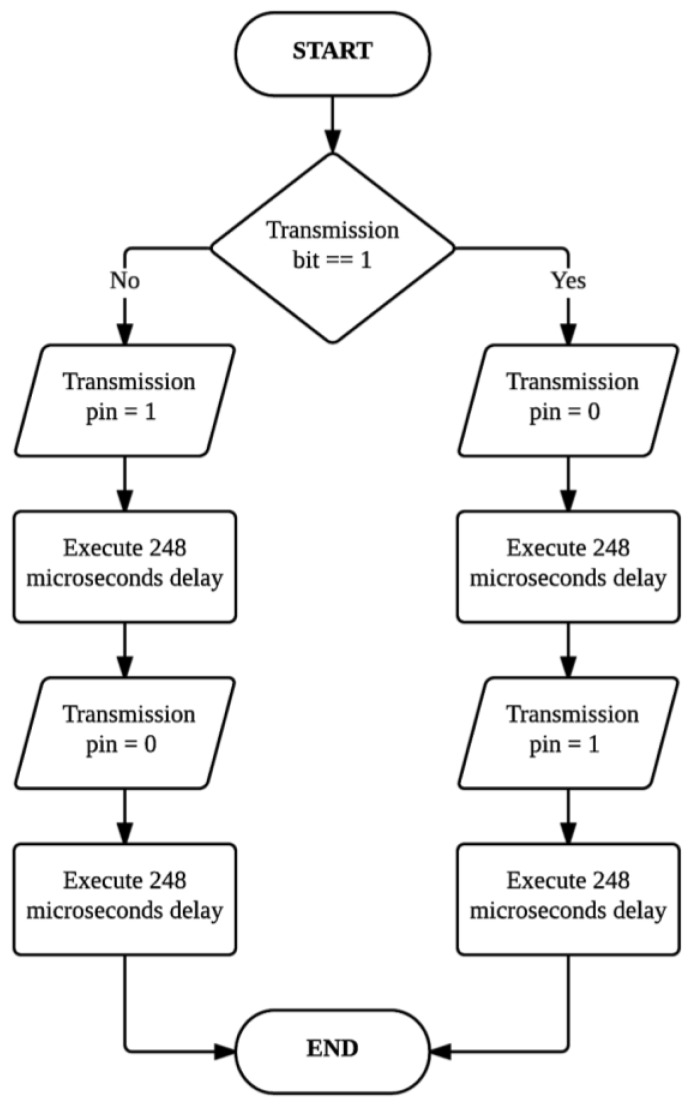
The Manchester encoding subroutine of the transponder data carrier.

If a binary one needs to be transmitted, the output pin of the microcontroller will output a binary zero for 248 microseconds and a binary one for another 248 microseconds. This results in a transmission rate of 2.015 kHz (496 μs). Figure 7 shows the Manchester encoding subroutines that are implemented.

**Figure 8:**
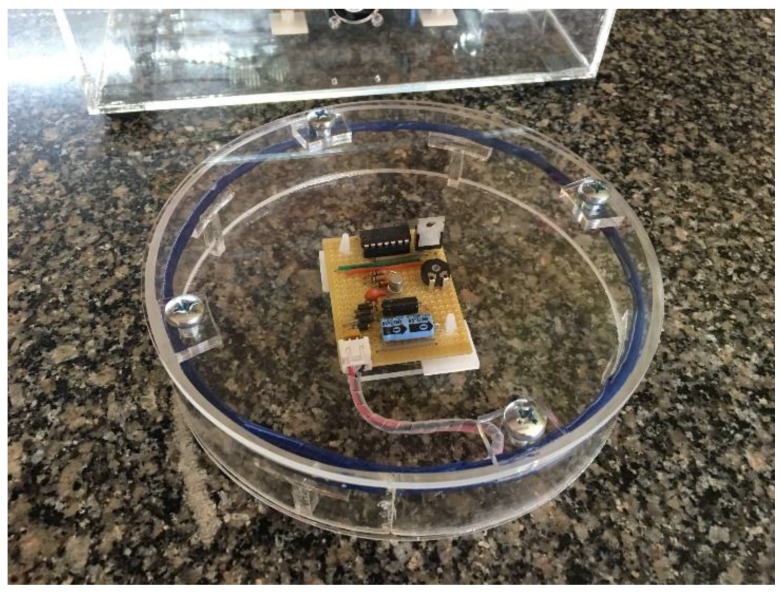
The circular coil RFID transponder.

**Figure 9:**
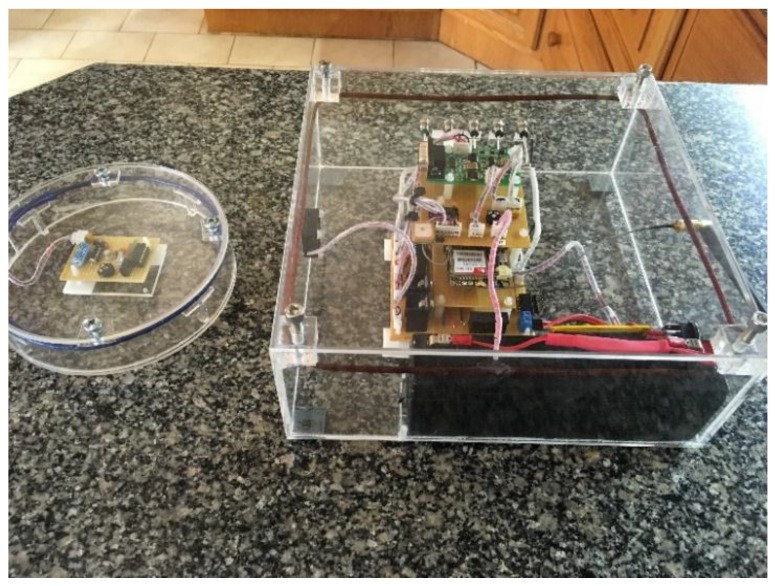
The main system and the RFID transponder with a circular antenna.

The software routines that are implemented on the low-power microcontroller required an accurate clock rate in order to execute optimally. A 4 MHz internal oscillator is used to ensure that the delays could be accurately calculated. It is found that, although the microcontroller can operate at low voltages, the clock speed of the microcontroller is not operating at the speed required to produce an accurate delay. This implies that although the microcontroller is activated at low voltage levels, communication between the reader and transponder would not succeed due to an inaccurate transmission rate. Figure 8 and Figure 9 show the main system, as well as the RFID transponder with the circular coil.

## 5. Results

### RFID Transponder Power Transfer

Table 6 below shows the practical values of the antenna inductance based on measurements made by an LCR meter.

**Figure 10:**
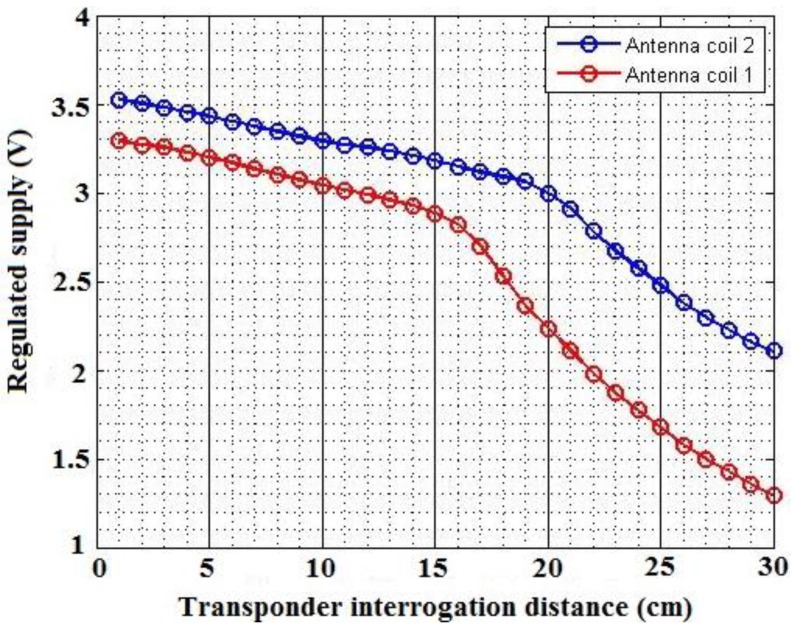
The voltage regulation with regards to the interrogation distance.

In order to measure the power levels of the transponder, two boxes are used as antenna mounts. The reader antenna is mounted to one box in an upright position, and the transponder antenna is mounted to a second box, also in an upright position. This allowed for the two antennas to be oriented in parallel, which allowed for maximum power transfer. The performance of the regulated power supply for the RFID transponder can be viewed in Figure 10. The result shows the regulation of a stable DC power supply. The configuration of the LC tank allows for adjustments to be made. Since the inductance values did not match perfectly, the capacitor values are adjusted with the use of Equation (3). The operating frequency of the reader is therefore still matched in order to achieve optimal resonance on the transponder side. From Figure 10, it can be seen that the voltage regulator circuit, together with the rectifier circuit, performed as expected. The voltage is successfully limited when the range between the transponder and reader is decreased. An exponential increase is limited to a linear increase in voltage.

**Figure 11:**
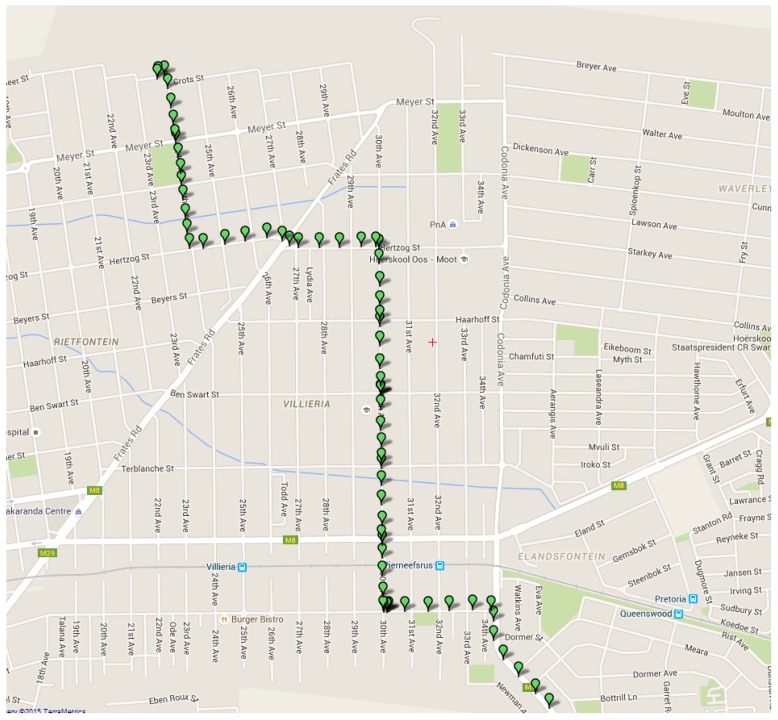
The retrieval, processing, and display of location information.

**Figure 12:**
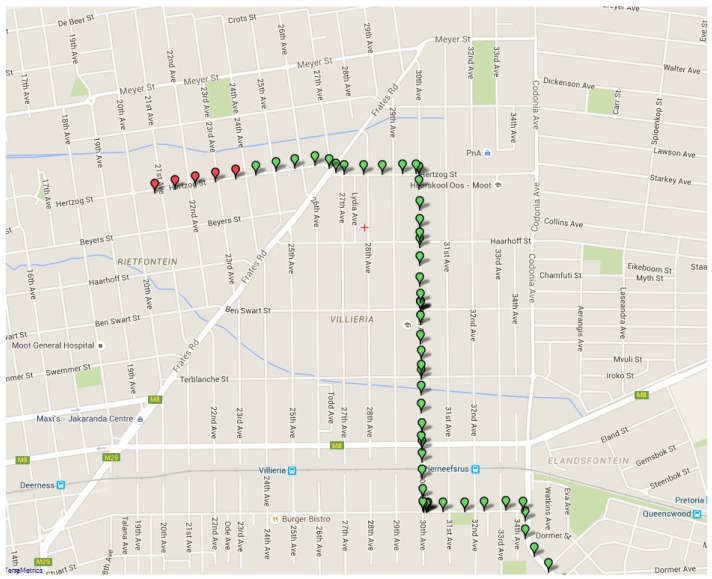
The straight-line prediction of the vehicle.

Figure 11 below shows the user interface and how location data is displayed. The location data is retrieved from the database system and processed on the interface side. From Figure 11 it can be seen that vehicle location data is successfully obtained and stored in the cloud. The data, which is represented by green markers, is also accurately displayed on the Google Maps interface. The estimation algorithm also has the ability to give straight-line vehicle positions, based on the location history of the vehicle. This can be viewed in Figure 12 (red markers are used for the estimation data). One observation that can be made from Figure 11, which is the fact that discrete data points are displayed. This is because location data is first retrieved and then transmitted via the GSM network to the database system. The transmission phase is first completed before new data is acquired. The estimation algorithm only has the ability to perform straight-line predictions in the case where the GPS module is switched off. This is because the system does not make use of any external sensors, such as an accelerometer, in order to determine if a vehicle has changed course or speed. Only the history of the vehicle position is used, which implies that a future change of direction cannot be predicted.

**Figure 13:**
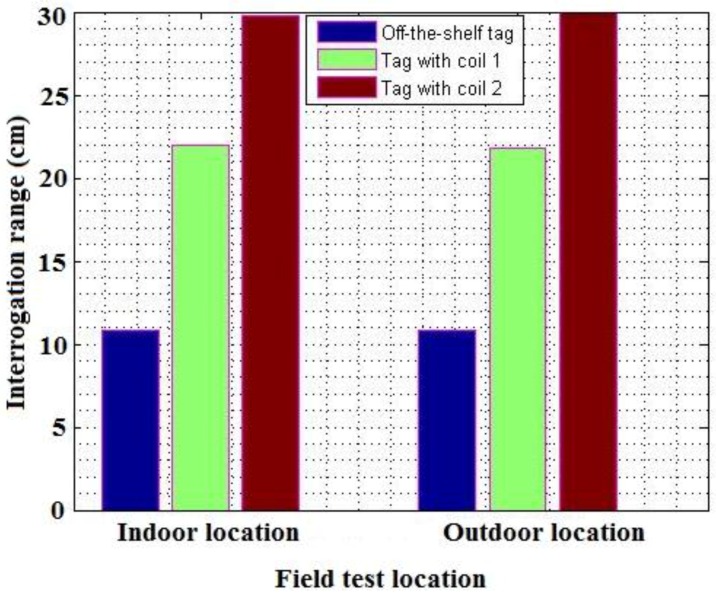
The different interrogation ranges obtained in an indoor and outdoor location.

From Figure 13 below, a clear comparison can be seen between the different transponders used and designed. The two designed transponders excelled in their interrogation range. The transponder with the circular coil did not achieve the specified read range. The transponder with the larger, square loop antenna did manage to achieve the specified interrogation range. The results also show a comparison between the performance of the transponders with regards to an indoor and outdoor location. It is clear that a good antenna design has a significant impact on the performance of a transponder with regards to the maximum read range that can be obtained. The primary constraint in RFID systems is the amount of power that can be delivered to a passive transponder over a distance. The delivered power decreases exponentially when the distance between a reader and transponder increases. By increasing the size of the transponder antenna, more magnetic flux can be intercepted by the coil, which implies that a greater voltage is induced over the antenna. It should be noted that there is a limit to the size of the transponder antenna. One primary factor that directly affects the read range, is the Q-factor of the antenna circuit. A higher Q-factor results in an increase in the maximum read range. The Q-factor is directly affected by the inductance of the antenna, and the internal resistance of the antenna. An increase in the antenna resistance results in a drastic decrease in the quality of the antenna. This therefore implies that an optimal inductance value is present for low-frequency RFID systems. In order to increase the inductance of a coil, more windings should be added. This however increases the resistance of the antenna, which is greater than the inductance value. For optimal performance, a balance should be found between the size of the antenna coil, and the inductance value of the coil.

## 6. Future Works

The primary objective of the research project is the design of an accurate vehicle location system with the integration of various independent technologies. Some level of prediction is added to the system, but it is believed that this area of the system can be greatly improved. During the implementation phase of the system, focus is placed on the design of an RFID transponder, which is mainly hardware design.

The RFID transponder is successfully implemented, and acceptable results are achieved. One possible suggestion is that the project should be adapted for the high frequency spectrum (or even the UHF spectrum), since a low frequency design is successfully completed. This would result in greater read ranges, and a much faster rate of data transfer would be achieved.

It is, therefore, believed that the focus of the project should now be shifted to the implementation of prediction models, in order to allow for the accurate prediction of vehicle routes and locations. This can be done with the design and implementation of Hidden Markov Models (HMM), which provides static predictions of routes, as well as destinations. Not only does this contribute to the prediction side of the project, but it also shifts the technical design focus of the project to expand the focus on the software side.

## 7. Conclusions

The main objective of this project was to design and implement an accurate vehicle location system that makes use of various independent technologies, such as GPS, GSM, and RFID. These standalone technologies were integrated into a single system that allowed for the locating and tracking of a vehicle in normal and complex environments, based on information that was retrieved and stored by the system. The three main subcomponents that were addressed were the acquisition of location data, the transmission and reception of location data, and the remote storage and analysis of vehicle location information.

The design included RFID antenna design, wireless power transfer and conditioning, modulation schemes, data encoding schemes, and software sequencing procedures. Additional technical challenges included the interfacing of independent technologies to a control unit, in order to form a complete autonomous system. The focus of the project is on the concept known as the Internet of Things. The importance and significance of the *IoT* concept was clearly seen with the incorporation of a GSM system that facilitated the wireless transfer of data. The transmitted data was stored on a database system, which allowed for the implementation of a graphical user interface. The database was managed with PHP scripts, which facilitated the storage and retrieval of data from the database system. The purpose of the interface was to display the location data of the vehicle in such a way that the vehicle can be easily tracked if necessary. The GUI was implemented as a desktop application, with the use of the Microsoft .NET platform. An estimation algorithm was implemented on the software side of the interface in order to add some level of prediction to the system. In general, it was found that a vehicle could be successfully tracked with the use of GPS and RFID technology. The use of GSM technology allowed for the transmission of data to the cloud for storage and processing. The graphical user interface assisted in the task to accurately display the data on a Google Maps interface.

Two RFID transponders were designed and implemented. It was found that a larger antenna design was generally considered better with regards to the interrogation distance. Furthermore, by implementing a larger antenna circuit on the RFID reader side, the read range was greatly enhanced. This proves that a maximum read range can be achieved by a good antenna design on both the reader and transponder side. An increase in the size of the RFID reader antenna, allowed for a greater electromagnetic field to be generated. By increasing the antenna size of the RFID transponder, more flux lines can be intercepted and utilized, which implies that a greater voltage is induced across the transponder antenna. The designed RFID transponders achieved a much greater interrogation range than the existing transponders in the market. It was also found that external elements have a minimal effect on the performance of low-frequency RFID applications. One observation that was made, was that low-frequency RFID designs might not be suitable for high-speed applications. This is proven by the fact that most RFID applications in the transport sector make use of the UHF band or microwave band. Nevertheless, an RFID transponder was successfully designed and tested and an average read range of 30 cm was achieved.

Emphasis is being placed on the development of autonomous systems for vehicles. A wide variety of problems need to be addressed in this field and by using and integrating various existing independent technologies, solutions can be found to problems that would hamper the effectiveness of autonomous systems. It is believed that the solution presented in this article can be implemented in the field of autonomous assistance systems. The use of RFID, GPS, and GSM technologies as a combined strategy for sensor inputs presents a unique opportunity for the development of sophisticated autonomous driver assistance systems. By utilizing different sensory inputs, undesirable effects such as fading GPS signals or multipath signals in city centers can be limited or even prevented.

## Figures and Tables

**Table 1:** RFID reader specifications.

Specification	Specification Value
Supply voltage	5.5–15 V
Operating current	38 mA
Operating temperature	0 °C to 85 °C
Maximum antenna voltage	250 V peak-to-peak
Maximum antenna current	200 mA

**Table 2:** The AT commands used for data transmission and reception.

Command Number	AT Command	General/GPS/GSM
1	AT	General
2	AT + CBC	General
3	AT + CGPSPWR = 1	GPS
4	AT + CGPSPWR = 0	GPS
5	AT + CGPSSTATUS?	GPS
6	AT + CGPSINF = 32	GPS
7	AT + CSTT	GSM
8	AT + CIICR	GSM
9	AT + CIFSR	GSM
10	AT + CIPSTART	GSM
11	AT + CIPSEND	GSM
12	AT + CIPCLOSE	GSM

**Table 3:** The internal parameters that are present in the LC tank.

Internal Parameters	Units of Measurement
Radiation resistance	Ohm (Ω)
DC resistance	Ohm (Ω)
Skin effect resistance	Ohm (Ω)
Wire inductance	Henry (H)
Loop inductance	Henry (H)
Parasitic capacitance	Farad (F)

**Table 4:** The design parameters of the circular coil antenna.

Antenna Parameters	Parameter Values
Wire diameter	0.2 mm
Coil diameter (*a*)	160 mm
Coil width (*b*)	2 mm
Coil height (*h*)	2 mm
Number of windings (*N*)	60

**Table 5:** The design parameters of the square coil antenna.

Antenna Parameters	Parameter Values
Wire diameter	0.4 mm
Coil diameter (*a*)	130 mm
Coil width (*b*)	3 mm
Coil height (*h*)	3 mm
Coil inductance	1 mH
Number of windings (*N*)	?

**Table 6:** The theoretical and practical antenna parameters.

Antenna Parameters	Circular Coil	Square Coil
Calculated theoretical inductance (mH)	2.862	1
Measured inductance (mH)	2.695	1.3
Calculated tuning capacitance value (pF/nF)	531 pF	1.52 nF
Capacitance value required based on the practical inductance value (pF/nF)	560 pF	1.82 nF
